# Evaluation of pediatric FMF patients with biallelic pathogenic exon 10 variants: focus on chest pain

**DOI:** 10.1007/s00431-026-06785-x

**Published:** 2026-02-11

**Authors:** Eray Tunce, Sıla Atamyıldız Uçar, Ekin İlayda Çağlar, Neslihan Kara Çanlıoğlu, Betül Sözeri

**Affiliations:** 1 Department of Pediatric Rheumatology, Ümraniye Training and Research Hospital, University of Health Sciences, İstanbul, Türkiye; 2 Department of Pediatrics, Ümraniye Training and Research Hospital, University of Health Sciences, İstanbul, Türkiye

**Keywords:** Chest pain, Colchicine resistance, FMF, Pediatric, Pleuritis, Serositis

## Abstract

Chest pain occurs in approximately one-quarter of patients with FMF, the most common monogenic autoinflammatory disease characterized by recurrent attacks of fever and serositis. This study evaluated a large, genetically homogeneous pediatric FMF cohort carrying biallelic pathogenic exon 10 MEFV variants to describe their demographic, clinical, and genetic characteristics, and to compare patients with and without chest pain to better define this manifestation within the FMF spectrum. This cross-sectional single-center study included 918 pediatric FMF patients with biallelic pathogenic *MEFV* exon 10 variants followed between June 2016 and February 2025. Demographic, clinical, and genetic data were collected retrospectively, and comparative analyses were performed between patients with and without chest pain. The cohort included 456 females (49.7%), with a median age of onset of 4.0 years and diagnosis age of 6.0 years. The most common genotype was M694V/M694V (48.9%), and 12.4% had colchicine resistance. Chest pain occurred in 218 (23.7%) patients, all pleuritic in origin, with no pericarditis. Those with chest pain had higher annual attack frequency, more abdominal pain (95% vs. 87%, *p* = 0.001) and greater colchicine resistance (21.1% vs. 9.7%, *p* = 0.001). The M694V/M694V genotype was significantly more frequent in those with chest pain (*p* = 0.017). Among patients presenting with chest pain, radiologically confirmed pleural effusion was detected in 5% during at least one of their evaluated attacks, predominantly among those with colchicine resistance.

*Conclusion*: In this large genetically homogeneous pediatric FMF cohort, chest pain was a frequent pleuritic manifestation. Patients with chest pain showed higher attack frequency and greater colchicine resistance, particularly among M694V homozygotes. Although rare, detected pleural effusion in patients with chest pain was associated with a significantly higher rate of colchicine resistance.

**Table:** 

**What is Known:** • *Chest pain is a recognized but variably reported manifestation of FMF, usually pleuritic in origin.* • *The M694V homozygous genotype is associated with more severe inflammatory phenotypes.*
**What is New:** • *In a large genetically homogeneous pediatric cohort, chest pain occurred in nearly one-quarter of patients and was linked to higher attack frequency.* • *Chest pain was strongly associated with colchicine resistance and more frequent in M694V/M694V patients. Additionally, a high frequency of colchicine resistance was observed in patients with detected pleural effusion.*

**What is Known:**

• *Chest pain is a recognized but variably reported manifestation of FMF, usually pleuritic in origin.*

• *The M694V homozygous genotype is associated with more severe inflammatory phenotypes.*

**What is New:**

• *In a large genetically homogeneous pediatric cohort, chest pain occurred in nearly one-quarter of patients and was linked to higher attack frequency.*

• *Chest pain was strongly associated with colchicine resistance and more frequent in M694V/M694V patients. Additionally, a high frequency of colchicine resistance was observed in patients with detected pleural effusion.*

## Introduction

Chest pain, most commonly resulting from pleural and occasionally pericardial involvement, is among the most prominent manifestations of familial Mediterranean fever (FMF). FMF is the most common monogenic autoinflammatory disease characterized by recurrent, self-limited episodes of fever and serositis affecting the peritoneum and pleura, as well as synovial inflammation of the joints [1]. The disease is caused by mutations in the *MEFV* gene, located on chromosome 16p13.3, which encodes the pyrin protein, a key regulator of the innate immune response [2]. Pathogenic variants in *MEFV* lead to uncontrolled interleukin-1β–mediated inflammation, resulting in periodic attacks of fever and pain [2, 3]. Among *MEFV* variants, M694V has been linked to a more severe clinical expression and a higher risk of amyloidosis [4, 5].

Clinically, FMF presents with recurrent inflammatory attacks that differ in severity, duration, and the predominant site of serosal involvement [6]. Abdominal pain and fever are the most frequent symptoms, whereas chest pain occurs less often but represents an important component of the disease’s inflammatory spectrum [7]. Chest pain in FMF is typically pleuritic in nature and most commonly results from pleural inflammation [8]. Pericarditis may also cause chest pain, although it remains a rare manifestation [8]. Despite being one of the characteristic manifestations of FMF, the frequency of chest pain and clinical associations vary considerably across different cohorts, ranging from 14 to 40% [9–11]. Recent studies suggest that chest pain may be linked to specific *MEFV* genotypes, particularly M694V, and may reflect a more severe inflammatory phenotype [11].

To date, studies investigating chest pain in FMF have mostly included genetically heterogeneous cohorts or a limited number of patients. The present study represents one of the largest single-center cohorts to date consisting exclusively of pediatric patients carrying biallelic pathogenic variants in exon 10 of the *MEFV* gene. In this study, we aimed to describe the demographic, clinical, and genetic characteristics of pediatric FMF patients carrying biallelic pathogenic variants in exon 10 of the *MEFV* gene and to compare clinical and genetic features between patients with and without chest pain to better characterize this manifestation within the FMF spectrum.

## Materials and methods

### Patients

This cross-sectional study included pediatric patients diagnosed with FMF carrying biallelic pathogenic variants in exon 10 of the *MEFV* gene and were followed at our tertiary pediatric rheumatology clinic between June 2016 and February 2025. For the diagnosis of FMF, we followed the new Eurofever/PRINTO classification criteria [12] for hereditary recurrent fevers. A total of 918 pediatric FMF patients who attended regular follow-up visits at three-month intervals for at least six months were included in the study.

Demographic data, family history, attack characteristics, *MEFV* genotype results, and treatment status were collected from the patients’ medical records. In addition to the overall cohort evaluation, detailed comparative analyses were performed based on the presence or absence of chest pain attacks. In pre-verbal children, chest pain was inferred from objective signs of pleuritis, including radiologically confirmed pleural effusion, respiratory distress, or splinting, accompanied by acute phase reactant elevation.

In accordance with our center's standard clinical protocol, all patients presenting with chest pain underwent an initial evaluation with posteroanterior and lateral chest radiographs and electrocardiography to exclude other alternative etiologies. Imaging records, together with echocardiographic and thoracic ultrasonographic examinations performed when clinically indicated, were reviewed to differentiate pleural and pericardial involvement. Conventional Sanger sequencing of the *MEFV* gene was employed as the reference method for genetic diagnosis. Colchicine resistance was defined as having ≥ 1 attack per month for at least three consecutive months despite receiving the maximum tolerated colchicine dose, in accordance with the 2024 EULAR/PReS FMF management recommendations [13].

### Study approval

The study was approved by the Institutional Ethics Committee of Ümraniye Training and Research Hospital. (Approval date: 10/04/2025, Number: B.10.1.TKH.4.34.H.GP.0.01/113). The study was conducted in accordance with the principles outlined in the Declaration of Helsinki. Because of the retrospective design of the study, the ethics committee waived the requirement for informed consent.

### Statistical analysis

Statistical analyses were conducted using IBM SPSS Statistics, version 30 (IBM Corp., Armonk, NY, USA). The Kolmogorov–Smirnov test was used to analyse the distribution of variables. Descriptive statistics were expressed using proportions, medians, and interquartile range (IQR; 25th-75th percentiles) values as appropriate. For comparisons between two independent groups with non-normally distributed continuous variables, the Mann–Whitney U test was used. Categorical variables were compared using the Chi-square test or Fisher’s exact test where appropriate. A *p*-value of less than 0.05 was considered statistically significant.

## Results

The study cohort consisted of 918 pediatric patients, including 456 (49.7%) females. The median (IQR; 25–75) ages at disease onset and at diagnosis were 4.0 (2.0–6.9) and 6.0 (3.0–9.0) years, respectively, with a median diagnostic delay 12 (6–24) months. The median disease duration was 5.1 (2.3–8.8) years, and the median age at the last visit was 12.8 (8.8–16.5) years. Parental consanguinity was present in 25.1% of patients, while 55.3% reported a family history of FMF. The median annual attack frequency before diagnosis was 12 (9–12), and the median duration of attacks was 3 (2–3) days. The main clinical findings of 918 patients were abdominal pain (88.9%), fever (86.4%). Amyloidosis was found in three (0.3%) patients. Colchicine resistance was detected in 12.4% of the cohort. Detailed clinical features are summarized in Table 1.

**Table 1:** Clinical differences between pediatric FMF patients presenting with and without chest pain

	Total Patients *n *(%), 918 (100)	With Chest Pain 218 (23.7)	Without Chest Pain 700 (76.3)	*p*-value
Sex, female^α^	456 (49.7)	108 (49.5)	348 (49.7)	0.964
Age at disease onset, years^β^	4 (2, 6.9)	4 (2, 7)	4 (2, 6.5)	0.345
Age at diagnosis, years^β^	6 (3, 9)	6 (4, 9)	5.2 (3, 9)	0.252
Diagnostic delay, months^β^	12 (6, 24)	12 (7, 24)	12 (6, 24)	0.080
Attack duration, days^β^	3 (2, 3)	3 (3, 3)	3 (2, 3)	0.348
Annual attack frequency before diagnosis^β^	12 (9, 12)	12 (12, 14.3)	12 (9, 12)	*0.034*
Parental consanguinity^α^	230 (25.1)	29 (22.5)	181 (25.9)	0.315
Family history of FMF^α^	508 (55.3)	133 (61.0)	375 (53.6)	0.054
Fever^α^	793 (86.4)	196 (89.9)	597 (85.3)	0.082
Abdominal pain^α^	816 (88.9)	207 (95.0)	609 (87)	0.001
Arthralgia^α^	489 (53.3)	124 (56.9)	365 (52.1)	0.221
Arthritis^α^	187 (20.4)	41 (18.8)	146 (20.9)	0.512
Pleural effusion^α^	11 (1.2)	11 (5.0)	0	-
Myalgia^α^	368 (40.1)	96 (44.0)	272 (38.9)	0.173
Exertional leg pain^α^	184 (20.0)	44 (20.2)	140 (20.0)	0.953
Amyloidosis^α^	3 (0.3)	0	3 (0.4)	-
ELE^α^	145 (15.8)	37 (17.0)	108 (15.4)	0.585
Diarrhea^α^	110 (12.0)	34 (15.6)	76 (10.9)	0.060
Vomiting^α^	83 (9.0)	18 (8.3)	65 (9.3)	0.644
Constipation^α^	36 (3.9)	12 (5.5)	24 (3.4)	0.168
Splenomegaly^α^	28 (3.1)	10 (4.6)	18 (2.6)	0.131
PFMS^α^	9 (1.0)	2 (0.9)	7 (1.0)	-
Headache^α^	73 (8.0)	29 (13.3)	44 (6.3)	*0.001*
Colchicine resistance^α^	114 (12.4)	46 (21.1)	68 (9.7)	*0.001*

^α^n (%)

^β^IQR (25-75p)

*ELE* Erysipelas-like erythema, *PFMS* Protracted febrile myalgia syndrome

The main genotypes in the cohort were M694V/M694V in 449 (48.9%) patients, M680I/M694V in 165 (18.0%), and M694V/V726A in 142 (15.5%) patients (Table 2).

**Table 2:** *MEFV* genotype distribution in FMF patients with and without chest pain

	Total Patients *n* (%), 918 (100)	With Chest Pain 218 (23.7)	Without Chest Pain 700 (76.3)	*p*-value
M694V/M694V	449 (48.9)	122 (56.0)	327 (46.7)	*0.036*
M680I/M694V	165 (18.0)	41 (18.8)	124 (17.7)	
M694V/V726A	142 (15.5)	29 (13.3)	113 (16.1)	
M694V/R761H	39 (4.2)	5 (2.3)	34 (4.9)	
M680I/V726A	39 (4.2)	7 (3.2)	32 (4.6)	
M680I/M680I	38 (4.1)	7 (3.2)	31 (4.4)	
V726A/V726A	20 (2.2)	2 (0.9)	18 (2.6)	
R761H/R761H	9 (1.0)	1 (0.5)	8 (1.1)	
M694I/M694V	7 (0.8)	2 (0.9)	5 (0.7)	
M680I/R761H	5 (0.5)	1 (0.5)	4 (0.6)	
V726A/R761H	3 (0.3)	1 (0.5)	2 (0.3)	
M694I/M694I	1 (0.1)	0	1 (0.1)	
M694I/V726A	1 (0.1)	0	1 (0.1)	

The overall genotype distribution differed significantly between patients with and without chest pain (Pearson χ^2^ = 22.2, df = 12, *p* = 0.036)

The frequency of the M694V/M694V genotype was significantly higher in the chest pain group compared with patients without chest pain (*p* = 0.017)

Patients were evaluated according to the presence of chest pain attacks. Chest pain was observed in 218 (23.7%) patients and was absent in 700 (76.3%). No patient had evidence of pericarditis, and all chest pain episodes were considered pleuritic in origin. The median annual attack frequency was significantly higher in those with chest pain (12 [12–14.3] vs. 12 [9–12]; *p* = 0.034). Abdominal pain was also more frequent among patients with chest pain (207 [95%] vs. 609 [87%]; *p* = 0.001). Headache was more common in patients with chest pain (29 [13.3%] vs. 44 [6.3%]; *p* = 0.001). Colchicine resistance was significantly higher in patients with chest pain (46 [21.1%] vs. 68 [9.7%]; *p* = 0.001). The overall genotype distribution differed significantly between patients with and without chest pain (*p* = 0.036). All three patients with amyloidosis were in the group without chest pain. No significant differences were found between the groups in other clinical or demographic characteristics. Among patients presenting with chest pain, radiologically confirmed pleural effusion was detected in 11 patients (5%) during at least one of their evaluated attacks; of these, 7 (63.6%) had colchicine resistance (Table 1). The frequency of the M694V/M694V genotype was significantly higher in patients with chest pain compared to others (*p* = 0.017) (Table 2).

Five patients experienced recurrent chest pain attacks without fever, abdominal pain, or arthritis. The characteristics of those five patients are presented in Table 3. Four were male and one was female. The age at disease onset ranged from 1 to 9.3 years, and the diagnostic delay ranged from 8 to 24 months. Attack duration varied between 2 and 7 days, while the annual attack frequency ranged from 3 to 24. A family history of FMF was present in two patients. Other disease-related clinical manifestations were observed in two patients, and colchicine resistance was noted in only one patient.

**Table 3:** Characteristics of FMF patients with isolated chest pain

	Patient 1	Patient 2	Patient 3	Patient 4	Patient 5
Gender	Male	Male	Male	Female	Male
Genotype	M694V/M694V	M694V/M694V	M694V/V726A	M694I/M694V	M680I/M694V
Age at disease onset, years^β^	6	9	9.3	2	1
Age at diagnosis, years^β^	8	10	10	3	2
Diagnostic delay, months^β^	24	12	8	12	12
Attack duration, days^β^	2	7	3	3	2
Annual attack frequency before diagnosis	12	3	12	8	24
Family history of FMF^α^	No	Yes	Yes	No	No
Other disease-related manifestations	None	Diarrhea, vomiting, arthralgia, myalgia, PFMS, ELE	Arthralgia, myalgia	None	Pleural effusion
Colchicine resistance	No	No	No	No	Yes

*ELE* Erysipelas-like erythema, *PFMS* Protracted febrile myalgia syndrome

## Discussion

This study provides a comprehensive overview of the clinical, genetic, and demographic features of a large single-center cohort of pediatric patients with FMF carrying biallelic pathogenic variants in exon 10 of the *MEFV* gene. Among 918 patients, classical FMF manifestations such as abdominal pain and fever were the most prevalent, whereas chest pain was documented in 23.7% of the cohort. None of the patients exhibited pericarditis, and all chest pain episodes were considered pleuritic in nature. Patients with chest pain had a higher annual attack frequency, a greater prevalence of abdominal pain and headache, and significantly higher rates of colchicine resistance compared with those without chest pain. The M694V/M694V genotype was notably more common among patients experiencing chest pain. Furthermore, pleural effusion was identified in a small subset of chest pain cases, most of whom had colchicine resistance. Together, these findings highlight the variability of serosal involvement in pediatric FMF and underscore the importance of genotype–phenotype correlations in understanding disease expression. This large, genetically homogeneous cohort provides valuable insight into both the overall clinical profile of pediatric FMF and the specific characteristics of chest pain attacks.

In our cohort, the median ages at disease onset and diagnosis were 4.0 and 6.0 years, respectively, with a median diagnostic delay of 12 months. These findings are largely consistent with previous reports from Türkiye [14–16]. Özbakır et al. [14] reported a median disease onset age of 4 years and a diagnosis age of 7 years in a biallelic cohort, while Öztürk et al. [15] found a similar onset around 4.6 years with an average diagnostic delay of approximately 18 months. Another Turkish national study [16] reported a median diagnosis age of 5.3 years. These observations indicate that FMF generally begins in early childhood and that variations in diagnostic timing may be influenced by differences in clinical presentation and disease awareness.

The median annual attack frequency before diagnosis was 12, which was higher than reported by Özbakır et al. [14] but similar to the larger national cohort reported by Öztürk et al. [15] (6 and 11 attacks per year, respectively). The median attack duration in our study was 3 days, consistent with findings from previous studies (3 days [14] and 2.7 days [16]). Overall, the attack frequency and duration in our cohort appear comparable to those observed in larger multicenter pediatric FMF studies, supporting the representativeness of our cohort within the national context [16].

In our cohort, the most common genotype was M694V/M694V, observed in 48.9% of patients. This rate was comparable to those reported in previous Turkish FMF studies (51.7% [16] and 73.1% [14]). Despite minor differences in frequency, the predominance of the M694V/M694V genotype remains a consistent finding across pediatric FMF cohorts in Türkiye.

Clinically, the most common manifestations were abdominal pain (88.9%) and fever (86.4%), followed by chest pain (23.7%) and arthritis (20.4%). These findings are consistent with previous pediatric FMF studies from Türkiye [15–17]. In our cohort, 12.4% of patients had colchicine resistance, closely aligning with the findings of the national TURPAID study, which reported a similar rate among patients with biallelic pathogenic *MEFV* genotypes [18].

Chest pain was observed in 23.7% of our patients, consistent with the rate reported in a national Turkish cohort [16] (26%) and slightly higher than that reported by Sunar-Yayla et al. [11] (14%). This difference may be attributed to the inclusion of all FMF patients, not only those with biallelic pathogenic variants, in that study [11]. None of our patients had pericarditis, and all chest pain episodes were attributed to pleuritic inflammation, whereas pericarditis was reported in 0.6% of cases in another Turkish cohort [15]. In our cohort, the median age at symptom onset and diagnosis did not differ between patients with and without chest pain (4 vs. 4 years and 6 vs. 5.2 years, respectively). In contrast, the study by Sunar-Yayla et al. [11] reported an earlier onset age in patients with chest pain (4 vs. 6 years). Similarly, the diagnostic delay was comparable between the groups (12 vs. 12 months), suggesting that chest pain phenotype is not necessarily associated with delayed recognition or diagnosis of FMF. The median annual attack frequency was significantly higher in patients with chest pain, consistent with that study [11], which also reported a greater attack count within the chest pain group. The M694V/M694V genotype was significantly more frequent among patients with chest pain, consistent with previous studies [11, 16] indicating that this variant is linked to a more severe inflammatory phenotype and a greater tendency toward thoracic serositis. In contrast, a previous study [11] reported a significantly lower frequency of chest pain among patients with the M694V/V726A genotype, whereas in our study, no significant difference was observed for this genotype. The V726A variant, which has generally been associated with milder disease expression [19], was not linked to an increased frequency of chest pain in our cohort, supporting the notion that this allele contributes to a less severe serositis phenotype compared with M694V.

Our cohort showed significantly higher rates of abdominal pain among patients with chest pain, which differs from previous study [11] on this manifestation, possibly reflecting a broader serosal inflammatory tendency.

Chest pain is also one of the clinical features included in the TURPAID colchicine resistance score, further supporting its association with disease severity [18]. In our study, the rate of colchicine resistance was markedly higher among patients with chest pain (21.1% vs. 9.7%). Similarly, Sunar-Yayla et al. [11] reported a higher rate of colchicine resistance in the chest pain group (7.4% vs. 1.5%). However, their cohort included patients with all genotypes, and overall colchicine resistance rates were lower than in our study. Since our cohort consisted exclusively of patients with biallelic pathogenic genotypes, both groups demonstrated higher resistance rates overall. Nevertheless, in both studies, the significantly higher colchicine resistance observed in patients with chest pain suggests that this manifestation may reflect a more severe or treatment-refractory inflammatory course. Notably, pleural effusion was detected in 11 patients (5%) with chest pain, and 7 of them (63.6%) exhibited colchicine resistance. While the presence of chest pain alone was associated with a higher rate of colchicine resistance in line with previous studies [11], our findings further may highlight that patients presenting with both chest pain and pleural effusion had an even greater tendency toward colchicine resistance, suggesting a more intense inflammatory involvement in this subgroup. However, given the limited number of patients in this subgroup, these findings require validation in larger, multicenter cohorts.

Although chest pain is among the three most common symptoms included in the FMF diagnostic criteria, only five patients in our cohort experienced isolated chest pain attacks without fever, abdominal pain, or arthritis. Two of these patients carried the M694V homozygous genotype. Additionally, one patient had pleural effusion accompanied by colchicine resistance. However, no marked clinical differences were observed between these patients and the overall cohort. Although the small number of patients limits definitive conclusions, the presence of isolated chest pain, as opposed to chest pain accompanied by other typical FMF symptoms, does not appear to substantially alter the overall disease manifestations or severity, suggesting that thoracic pain in FMF typically coexists with systemic inflammatory activity.

This study has certain limitations. Its retrospective and single-center design may limit the generalizability of the findings. In addition, clinical data were obtained from patient records, which may have introduced recall bias. Another limitation involves the timing of radiological imaging. Since this was a retrospective real-life study, chest X-rays were performed at the time of patient presentation, which varied in duration from symptom onset. Consequently, transient or late-developing pleural effusions might have been missed in patients imaged only during the early hours of an attack. Therefore, the reported 5% frequency of pleural effusion likely represents an underestimation of the true prevalence.

In conclusion, in this large, genetically homogeneous cohort of pediatric FMF patients carrying biallelic pathogenic exon 10 variants, chest pain was a frequent manifestation, predominantly of pleuritic origin. Patients with chest pain exhibited higher attack frequency and a greater prevalence of colchicine resistance, particularly among those with the M694V homozygous genotype. Although rare, detected pleural effusion in patients with chest pain was associated with a significantly higher rate of colchicine resistance. Overall, this study adds valuable insight into the clinical spectrum of pediatric FMF by characterizing one of the largest genetically confirmed biallelic exon 10 cohorts reported to date.

## Data Availability

Raw data cannot be shared due to the protection of patients’ privacy. All data relevant to the study are included in the article. Please contact the corresponding author.

## Abbreviations

FMF: Familial Mediterranean Fever
*MEFV*: Mediterranean fever gene
IQR: Interquartile range
PRINTO: Paediatric Rheumatology International Trials Organisation
EULAR: European Alliance of Associations for Rheumatology
PReS: Paediatric Rheumatology European Society
